# Evolution of Pathological Techniques for the Screening of Cervical Cancer: A Comprehensive Review

**DOI:** 10.7759/cureus.60769

**Published:** 2024-05-21

**Authors:** Priya B Chatterjee, Snehlata R Hingway, Kishor M Hiwale

**Affiliations:** 1 Pathology, Jawaharlal Nehru Medical College, Datta Meghe Institute of Higher Education and Research, Wardha, IND

**Keywords:** artificial intelligence, human papillomavirus (hpv), colposcopy, cervical cancer vaccines, uterine cervical cancer, liquid-based cytology, conventional papanicolaou (pap) smear, cervical cancer screening

## Abstract

The evolutionary journey of cervical cancer screening has been a major medical success story, considering the substantial role it has played in dwindling the disease burden. Through sustained collaborative efforts within the medical community, significant advances have been made from the humble yet path-breaking conventional Pap smear to the current automated screening systems and human papillomavirus (HPV) molecular testing. With the integration of artificial intelligence into screening techniques, we are currently at the precipice of circumventing the pitfalls of manual cytology readings and improving the efficiency of the screening systems by a significant margin. Despite the technological milestones traversed, the high logistics and operational cost, besides the technical know-how of operating the automated systems, can pose a major practical challenge in the widespread adoption of these advanced techniques in cervical cancer screening programs. This would suggest the need to adopt strategies that are tailored to the demands and needs of the different settings keeping their limitations in mind. This review aims to take the reader through the entire evolutionary journey of cervical cancer screening programs, highlight the individual merits and demerits of each technique, and discuss the recommendations from the major global guidelines.

## Introduction and background

The WHO data from the year 2022 revealed cervical cancer as the fourth most common cancer in women, with around 6,60,600 new cases detected in the year 2022 [1]. A strong predilection was observed toward an increased prevalence rate of cervical cancer rates in Sub-Saharan Africa, Central America, and South-East Asia [1]. Further, a significantly higher quantum of disease-associated morbidity and mortality (94% of the 3,50,000 deaths caused by cervical cancer) was observed in low- and middle-income countries in the year 2022 [1]. In India, cervical cancer contributes to approximately 6-29% of all cancers in women [2]. The cervical cancer country profile by the WHO in 2021 put the crude incidence at 18.7 per 1,00,000 women, and the mortality-to-incidence ratio at 0.62 in the year 2020 [2]. The reported age-adjusted incidence rate of cervical cancer in India varied widely, the highest being 23.07/1,00,000 in Mizoram state and the lowest in Dibrugarh district with 4.91/1,00,000 [2].

Pertinently, cervical cancer can be effectively prevented with robust screening strategies [3]. Cervical cancer screening methods have undergone a significant evolution over the years, facilitated by sustained scientific and technological advancements. Despite the advances, in the past, the wide economic disparities between countries across the world may have lent to a significant skew in the disease and its associated mortality and morbidity distribution toward the low- and middle-income countries [1]. However, with continued advancements in screening strategies, there remains the possibility of the development and subsequent deployment of rapid, cost-effective, and sensitive cervical cancer screening techniques, which may potentially lower the quantum of the disease burden significantly in developing nations.

In this manuscript, we take a closer look at the evolution of the pathological screening techniques for cervical cancer as well as the newer emerging methodologies, including the integration of artificial intelligence (AI) in the screening techniques.

## Review

Developments in the era prior to the emergence of Papanicolaou (Pap) smear

The earliest recorded descriptions of cervical carcinoma in situ were provided by Sir John Williams in 1886 [4]. The histological details were illustrated by Thomas Cullen in the published text 'Cancer of the Uterus' in 1900 [4]. The concept of carcinoma in situ characterized by an intra-epithelial growth phase was developed further by Schauenstein, Pronai, Rubin, Schottlander, and Kermauner between 1908-12 [5]. This was later coined by Walter Schiller as 'pre-invasive carcinoma' [6]. Schiller defined it as "cytological atypia without evidence of invasion through the basement membrane" [7]. However, this terminology was later replaced by Dr. A.C. Broders with 'carcinoma in situ'. During this phase before the 1940s, the diagnosis was dependent heavily on tissue biopsies and subsequent microscopic examinations [8-10]. However, biopsies were largely performed only in the presence of a visible lesion, with any diagnosis based on premalignant lesions being a rarity. The scientific realization that pre-invasive abnormalities tend to pre-date cervical cancer by several years emerged around the 1940s. It gained traction when histological examinations of serial sections of 1200 clinically normal services revealed an incidence of 4% of pre-invasive lesions [11]. The awareness further intensified once the practice of histological evaluation of conization specimens was introduced for specimens showing abnormal cells in cytological examination. Furthermore, the notion of cervical cancer being a preventable disease was propagated first by Dr. Paul A. Younge, who was a protégé of Dr. Broders and ran a clinic for the diagnosis and treatment of cervical carcinoma in situ [12].

Emergence of Pap smear and subsequent improvements

Despite all the prior scientific endeavors, the credit for establishing cervical cancer as a preventable disease through effective screening strategy lay squarely with the Greek physician and physiologist, Dr. George Papanicolaou, as per popular medical literature [13]. He was a reluctant clinical practitioner, whose primary interests lay as a medical scientist. He emerged into prominence due to a technique developed by him in guinea pigs for obtaining vaginal contents and microscopically evaluating them to identify multiple cell types with various changes that could be correlated with their hormonal status [14,15]. On application of this technique in humans, he was able to recognize its value in cervical cancer detection [15]. However, when he presented his results in 1928 at the Third Race Betterment Conference in Battle Creek, Michigan, United States, his findings were not received well by the medical community [16]. In addition to his presentation being marred by inadequate photographs and typographical errors, the primary bone of contention among the pathologist community, for whom tissue diagnosis was at the core of any diagnosis, was their non-alignment with the notion that 'dead' cancer cells could have any diagnostic utility [16]. Simultaneously, Romanian pathologist, Dr. Aurel A. Babes had also presented similar findings in an article titled 'The diagnosis of cancer of the uterine cervix from smears', with pertinent differences from Dr. Papanicolaou being that he had obtained his samples from the cervix and that he had also emphasized the theory that invasive cervical carcinoma was preceded by a prolonged pre-invasive stage, which was not propagated during those years [13].

Despite the initial setback, Dr. Papanicolaou's work took off under the mentorship of Dr. Joseph Hinsey, who encouraged him to collaborate with gynecologist and pathologist Dr. Herbert Traut as well as gynecologist Andrew Marcetti [14]. Thay et al. evaluated the vaginal smears in all women admitted to the obstetrics and gynecology department and observed that cancers in asymptomatic and clinically normal women indemonstrable by biopsy could be detected through vaginal smears [14]. Their observations were published in a seminal article titled 'The diagnostic value of vaginal smears in carcinoma of the uterus' in the Journal of Obstetrics and Gynaecology in 1941 [14]. Detailed illustrations with the aid of Hashima Murayama (an expert illustrator who later worked with the National Geographic Society) and diagnostic criteria for cervical cancer were subsequently published in a monograph titled "Diagnosis of uterine cancer by the vaginal smear" [14]. Over the subsequent years, Dr. Papanicolaou further refined the technique by improving the fixation and staining properties of the smear to further optimize the detection of abnormal cells. His publications establishing the utility of exfoliative cytology for the detection of pre-cancerous/cancerous cervical lesions ushered in the era of cytopathology.

Among the subsequent attempts at improving upon the Papanicolaou method, Dr. J. Ernest Ayre's attempt at using a wooden spatula to directly scrape the cervix in 1948 stands out [14]. His selective scraping method often compared to a 'surface biopsy' of the cervix, aimed at scraping the circumference of the cervix and thereby obtaining cells that had not yet exfoliated [14]. The design of the spatula has remained the same to date, and his technique bore a lot of similarities with that of the technique developed by Dr Aurel A. Babes, with the exception that he had utilized platinum loops for obtaining the cervical samples [14]. However, Dr. Ayre convinced the medical community of the higher degree of sensitivity of his method and proposed it as the method of choice in detecting early microscopic disease. Subsequently, the 'Papanicolaou test' as it came to be known popularly, also came about to be endorsed and promoted by the American Cancer Society (ACS) and the National Cancer Institute (NCI) in the 1950s [17]. 

The next major milestone in this space was the introduction of the Clinical Laboratory Improvement Amendments (CLIA) in 1988 by the United States (US) Congress. This act was developed in response to the public outrage following the Wall Street Journal articles by Walt Bogdanich highlighting the grueling workload of the cytotechnologists, which often led to high rates of false negative results [13,18]. This law was aimed at regulating the workload of the cytotechnologists and laying down stringent quality control procedures to improve the testing outcomes. Another major fallout of the CLIA legislation was the emergence of multiple gynecologic cytology litigations, thereby bringing to light the need for better and advanced screening techniques [18]. Subsequently, various attempts were made to improve the devices for sample collection, including the introduction of brush devices for obtaining samples from the endocervix and transformation zones. However, it must be remembered that cytology-based cervical cancer screening remains highly subjective and provider-dependent, with a high degree of variations existing between laboratories and cytologists reporting the smears.

Nonetheless, to bring a certain uniformity in the system of cervical cancer reporting worldwide, the Bethesda system is used for reporting cervical cytology. It has three basic principles. The first is that the classification terminologies used should be uniform across the world, but at the same time, they should be mouldable to suit local population needs. Second, the cervical smear report should give clinically appropriate and relevant information to the treating clinician. The third and last principle is that the terminologies used in the report should be updated periodically so as to have a current understanding of cervical cancer. Moreover, the presence of inflammation-related debris in the smears, more so due to the high incidence of cervical inflammation in developing nations, is another significant confounding factor to be considered while reporting on the smears. Despite the best of quality control efforts, there remains a high incidence of false negative rates (14-35%), with the sensitivity and specificity of a quality assured smear for detecting cervical intraepithelial neoplasia (CIN) 2+ lesion being 60-95% [19,20].

Unfolding of the human papillomavirus (HPV) and cervical cancer link

The next major milestone in the evolution of cervical cancer screening was the discovery of the HPV and the establishment of its link with cervical cancer. The discovery of the HPV ensued closely on the heels of the enactment of CLIA legislation [21]. It was Dr. Harold zur Hausen who established the link between HPV and cervical cancer by isolating genotypes 16 and 18, for which he was awarded the Nobel Prize in 2008 [21]. To date, more than 450 genotypes of HPV have been identified, organized, and numbered based on genetic makeup. Low-risk or non-oncogenic HPV genotypes are 6, 11, 42, 43, and 44 while high-risk or oncogenic HPV types include 16, 18, 31, 33, 35, 39, 45, 51, 52, 56, 58, 59, 68, 73, and 82 [21]. Among all genotypes, HPV-16 followed by 18 is the most carcinogenic and is associated with more than 70% of squamous cell and adenocarcinoma of cervix [21]. Others like HPV-31, 33, 35, and 52 are considered medium risk and have a 5%-6% chance of developing cervical cancer [21]. The low-risk subtypes are occasionally found in cervical carcinomas. The virus usually infects the lining epithelium of the squamocolumnar junction and produces viral particles in the mature epithelial cells, hence causing disruption in normal cell cycle control and further promoting uncontrolled cell division, leading to genetic damage. Most of the HPV-induced cervical changes are transient and regress spontaneously within one to two years of exposure [21]. However, persistent infections with high-risk genotypes along with genetic predisposing factors like polymorphic genes of various major histocompatibility complexes, as well as polymorphism in the p53 gene involved in the clearance and maintenance of HPV infection, can often end up developing high-grade squamous intra-epithelial lesions, which in turn have a 0.5-1% per year risk of progression to invasive squamous cell carcinoma [21].

With improved comprehension of the HPV's biology and its link with cervical cancer, in the background of the emerging need to improve the reproducibility of the testing reports, the Bethesda reporting system was introduced in 1988 in Maryland, US, which proposed a two-tier system by dividing intra-epithelial lesions into high-grade squamous intra-epithelial lesions (HSIL) and low-grade squamous intra-epithelial lesions (LSIL) [18]. It also categorized equivocal atypia, i.e., neither non-neoplastic nor pre-neoplastic, with a qualifier of 'undetermined significance' [18]. Further, the results of the atypical squamous cell of undetermined significance (ASC-US)- LSIL Triage Study (ALTS) helped in addressing the issue of diagnosis of a large number of ASC-US cases and the variability in the management due to the lack of standard recommendations for this category [22]. This study established that HPV testing via Digene Hybrid Capture II (HC2) HPV test (Qiagen, Germantown, Maryland, US) was a very cost-effective way to manage ASC-US cytology cases. This led to the US Food and Drug Administration's (USFDA's) approval of the HC2 HPV test as a mandatory test for ASC-US cytology. Later, due to its cost-effectiveness, the HC2 HPV test was also recommended as an adjunct screening tool in 2003 for women of 30 years and above age group [23,24]. Subsequently, in 2014, based on the results of the ATHENA study, HPV testing alone without cytology was also approved by the USFDA as a primary screening option [25]. Also, it is proven that the positivity rate of CIN 2 has decreased in females with prior HPV vaccination, stronger for girls vaccinated at an early age. Based on feasibility, HPV testing has emerged as the most suitable test for screening women since it is to date the most accurate, reproducible, provider-independent test with a sensitivity of >90% and >95% to detect CIN 2+ and CIN 3+ lesions, respectively [26]. Though, less specific, considering the high negative predictive value of this test, it scores over cytology as the preferred screening test, keeping aside economic and logistics considerations [27,28].

Visualization methods for primary cervical cancer screening

Visualization with colposcope, a technique which was first described by Hans Hinselman from Germany in 1925, involves visualizing the entire cervix, with greater emphasis on inspection of two areas: the squamocolumnar junction and the transformation zone, since these represent areas at greatest risk of neoplasia [29]. Visual inspection with 3-5% acetic acid (VIA) functions by dehydrating the cells such that metaplastic cells/dysplastic cells/cells infected by HPV possessing a large dense nucleus will reflect light to produce acetowhite changes, a hallmark of cervical neoplasia [30]. Acetic acid can be replaced by Lugol's iodine to produce yellowish discolorations in neoplastic areas due to lack of glycogen in these cells, which is quite distinct from the normal cells where the adequate intracellular glycogen on coming in contact with Lugol's iodine produces a brownish-black discoloration [31]. These techniques were developed to identify ways to improve upon the pitfalls of the cytology techniques for cervical screening and have now emerged as more viable and better alternatives to cytology in resource-constrained settings [32].

Visualization techniques with colposcopy are quite cost-effective, safe, and simple, and hence can be performed by a wide range of medical service providers [32]. Further, there is a strong wall of evidence to attest to the safety and feasibility of these techniques, especially in resource-constrained settings. A comprehensive meta-analysis involving 57 studies utilizing VIA as the primary screening tool in high and low-income countries in both hospital and rural settings revealed a sensitivity of 80%, specificity of 92%, a positive predictive value of 10%, and a negative predictive value of 99% [33]. Moreover, VIA's accuracy was not affected by region, setting, population size, and training level of the service provider [33]. This high negative predictive value was also reproduced by another longitudinal study performed by Sankaranarayan et al., where among the 23,000 VIA-negative adult women, only 25 were diagnosed with cervical cancer in the subsequent eight years of longitudinal follow-up [28]. However, high false positives in regions where cervical inflammation rates are quite high along with the absence of a permanent image/pathology for documentation and the need for good lighting and adequate visual acuity of the service provider are some of the limitations of this technique [32].

Among the recent advances, portable digital colposcopes have been developed, which have the same features as regular colposcopes. In addition to the standard colposcopes, ultra-high-resolution digital images can be obtained. These images can be magnified to higher degrees than a conventional colposcope, hence giving better visualization of cervical cell morphology. As per a prospective study conducted by Thay et al. in HIV-positive and negative Cambodian women, HPV-positive females (self-swab method) underwent digital colposcopy (DC), which accurately diagnosed females with cervical dysplasia, and those positive cases were further confirmed on histopathology [34]. This study recommended large-scale screening programs since DC proved to be not only efficient and accurate but also cost-effective and can be used as a mass screening method for diagnosing cervical lesions early [34]. The classification of cervical neoplasms on colposcopy is given as binary classifications of high-risk vs. low-risk lesions. The high-risk lesions include CIN 2, CIN 3, and CIN 2+, and low-risk lesions include CIN 1 and CIN 1-. The lower anogenital squamous terminology (LAST) high-risk lesion includes HSIL, and the LAST low-risk lesion includes LSIL and normal lesions (LSIL-). Multiple systematic reviews have also attested to the better sensitivity and specificity of DC over VIA, primarily due to its magnification power [35]. Apart from this, DC provided the benefits of facilitating patient and provider education using the images as well as permanent documentation for records and quality control and for aiding in real-time telemedicine consultation with expert colposcopists [32].

Liquid-based cytology (LBC) and automation in cervical cancer screening

Attempts at automation in screening have been in progress since the 1950s to maximize screening efficiency. However, it was not until the FDA's approval of the PAPNET (Neuromedical Systems, Inc., Suffern, New York, US) (only for re-screening) and the AutoPap 300 QC Automatic Pap Screener System (Redmond, Washington, US) (for primary screening and re-screening) in the mid to late 90s that automated cervical cancer screening systems came to fruition [36-38]. Subsequently, LBC preparations came into the picture, which represented the first major improvement in the cervical screening sample preparation method in almost 50 years [39]. Instead of smearing the cells onto a glass slide, the cervical samples are washed into a liquid transport medium, which fixes, homogenizes, and rinses the cells [40]. Within the apparatus, a filter or a settling chamber is used to concentrate a thin layer of cells within a circular area or cell disc on the glass slide. LBC works on the principle that though the cells within the circular area in the glass slide may not represent the entire cell population collected, they represent a random portion of the sample, which is reproducibly representative of the entire cell population on a sampling device [40].

LBC scores over conventional Pap smears as it aims to optimize cell preservation to reduce the likelihood of getting unsatisfactory smears and decrease artifacts and debris like inflammatory cells, blood, and mucus, which may impede the microscopic readings, as well as reduce the turnaround time for reporting [41]. To date, two major LBC preparations have been approved by the FDA: ThinPrep (Cytyc Corporation, Boxborough, Massachusetts, US) in 1996, as an alternative to cervicovaginal smears in the US, followed by SurePath (Becton, Dickinson and Company, Franklin Lakes, New Jersey, US) in the year 1999 [41]. The American College of Obstetricians and Gynecologists has approved both methods for cervical screening. Despite the ambiguity over the superiority of LBCs over conventional cytology, the literature unanimously mentions that LBCs can be considered, overall, to be equally sensitive and specific compared to conventional cytology for the detection of CIN 2+ and worse lesions [42]. However, LBC implementation in screening programs in resource-constrained settings is laced with serious practical challenges due to LBCs not being cost-effective and considering the need for trained technicians and cytopathologists to interpret the results. In developed countries with well-established screening programs and wide availability of LBC, screening programs are the single most successful tool for cervical cancer prevention; however, biopsy remains the gold standard for confirmation of the abnormality detected in LBC [32].

Among the subsequent advancements in automation for cervical cancer screening, the USFDA approved location-guided screening techniques utilizing liquid-based preparations at the turn of this century [43]. These screening techniques utilize location-guided technology and proprietary algorithms to identify key areas on computerized images most likely to harbor abnormal cells, which are then later manually reviewed by cytologists [43]. Though the results from the initial studies were extremely encouraging, the same results have not been reported from studies performed outside the US, thereby limiting the acceptability of such techniques [43]. More recently, cervical cancer screening has seen the introduction of AI into the automated smear analysis system [44-46]. This system comprises five stages: image acquisition, preprocessing, segmentation, feature extraction, and classification. AI technology has been mainly applied in the segmentation and classification stages for the automatic analysis of cervical smears. Considering the importance of the accurate classification of abnormal cells, AI by improving the accuracy in cell classification and bypassing the requirement of professional degree of the observer, scores over manual cytology. Studies confirm that there was a significant improvement in the detection rate of CIN when also compared with the gold standard, cervical biopsy.

As per data studied with AI, there was an improvement in the rates of sensitivity and specificity in differentiating high-grade cervical lesions from low-grade cervical lesions, and thus it helped in reducing the rate of misdiagnosis [47]. Bao et al. revealed that the sensitivity to diagnose CIN 1 was 88.9% and specificity was 95.8%, for CIN 2, sensitivity was 90.10% and specificity was 94.40%, and for CIN 3, sensitivity was 90.90% and specificity was 94.40% [47]. Wong et al. also found the sensitivity and specificity to be >90% in their study conducted in 2019 to identify high-grade lesions with the application of AI [47]. Pathania et al. conducted a study using a deep learning (DL) algorithm combined with digital micro-holography, which showed excellent results (specificity and sensitivity 100%) in detecting HPV 16 and 18 [47]. Tian et al. studied the risk stratification model for cervical precursor lesions, which successfully predicted CIN 2 with an average accuracy probability score of 0.814 [47]. This method helped in effectively stratifying the risk of cervical lesions and also provided valuable triage strategies. Chankong et al. conducted a study on the segmentation of the cervical single-cell image into the nucleus, cytoplasm, and background [47]. This study showed an accuracy result of more than 93%. Shi et al. conducted a study to propose a method of cervical cell classification [47]. It concluded with a potential relationship between cervical cell images and improvement of classification performances with 98.3% accuracy, 99.80% sensitivity, and 99.60% specificity. With its powerful image analysis ability, AI has solved the problem of diagnosis of cervical cancer lesions by multiple colposcopy images.

Guidelines recommendations for cervical cancer screening

The current cervical cancer screening recommendations as mentioned by the US Preventive Services Task Force (USPSTF), 2018, the American Cancer Society (ACS), 2020, and the American College of Obstetricians and Gynecologists (ACOG), 2021, are summarized in Table 1 [48-50].

**Table 1:** The cervical cancer screening recommendations as mentioned by the USPSTF, 2018, the ACS, 2020, and the ACOG, 2021. USPSTF: United States Preventive Services Task Force; ACOG: American College of Obstetricians and Gynecologists; ACS: American Cancer Society; HPV: human papillomavirus; HIV: human immunodeficiency virus; CIN: cervical intraepithelial neoplasia; HSIL: high-grade squamous intraepithelial lesion; LSIL: low-grade squamous intraepithelial lesion

Target group	Screening test recommendation
Individuals at average risk	Age 21-29 years - ACOG and USPSTF guidelines: cytology alone every three years
	Age 30-65 years - ACOG guidelines: any of the three - cytology alone every three years or HPV testing alone every five years or cytology + HPV testing every five years; USPSTF guidelines: cytology + HPV testing every five years or HPV testing alone every five years
	Age greater than 65 years - ACOG guidelines: no screening after adequate negative prior screening results; USPSTF guidelines: cytology alone every three years
	ACS recommends any of the three: cytology alone every three years or HPV testing alone every five years or cytology + HPV testing every five years
Individuals with an immunocompromising medical condition (e.g., HIV infection, Solid organ transplantation)	Age 21 to 65 years - ACOG and ACS guidelines: cytology every year - after three consecutive annual normal cytology tests, screening should be every three years. USPSTF: no specific recommendations
Individuals with in-utero exposure to diethylstilbesterol	ACOG guidelines recommend annual cytology. USPSTF and ACS: no specific recommendations
Individuals with HPV vaccination	No changes to the screening approach as mentioned above
Individuals with a diagnosis of CIN 2+ (histologic HSIL) within the prior 25 years	Initial surveillance - HPV testing alone or cytology + HPV testing at 6, 18, and 30 months or cytology alone at 6, 12, 18, 24, and 30 months. Long-term surveillance - HPV testing alone or HPV + cytology testing every three years or cytology alone annually. Continue for more than 25 years from the time of initial CIN diagnosis even if age >65 years. Routine screening can resume after the post-treatment surveillance period

## Conclusions

In the continued search for refinement and improving the efficiency of cervical screening strategies, a significant journey has been traversed from the rudimentary yet seminal Pap smear to the current automated liquid-based systems and HPV testing. With the introduction of AI in cervical cancer screening, we are at an inflection point, where the pitfalls of manual cytology testing can be circumvented to achieve a high degree of reproducibility and effectiveness with a significantly curtailed turnaround time for reporting. However, the benefits of these advances are often offset by their high infrastructure and logistics costs, which poses a major practical challenge within resource-constrained settings like India. In such scenarios, tailored screening strategies utilizing techniques that walk a tightrope in terms of balancing cost-effectiveness with maintaining an acceptable accuracy of the readings, such as VIA and HPV, need to be adopted to reduce the disease burden. 
